# Supplementary material for: Resolving the full spectrum of human genome variation using Linked-Reads
Source: Genome Res. 2019 Apr;29(4):635–45. doi: 10.1101/gr.234443.118 (PMC6442396; doi:10.1101/gr.234443.118)
Supplement: Supplemental Material [file supp_gr.234443.118_Supplemental_longranger-2.2.zip › longranger-2.2.tar.bz2!/lr2.2-public/lib/stan/cmdstan/stan/doc/api/html/agrad_2fwd_2matrix_2log__softmax_8hpp_source.html]

Stan: src/stan/agrad/fwd/matrix/log\_softmax.hpp Source File


|  |  |
| --- | --- |
|  | Stan  2.5.0  probability, sampling & optimization |


- Main Page
- Related Pages
- Namespaces
- Classes
- Files

- File List
- File Members

All Classes Namespaces Files Functions Variables Typedefs Enumerations Enumerator Friends Macros Pages

- src
- stan
- agrad
- fwd
- matrix

log\_softmax.hpp

Go to the documentation of this file.

1 #ifndef STAN\_\_AGRAD\_\_FWD\_\_MATRIX\_\_LOG\_SOFTMAX\_HPP

2 #define STAN\_\_AGRAD\_\_FWD\_\_MATRIX\_\_LOG\_SOFTMAX\_HPP

3

4 #include <stan/agrad/fwd/fvar.hpp>

5 #include <stan/agrad/fwd/matrix/softmax.hpp>

6 #include <stan/math/matrix/Eigen.hpp>

7 #include <stan/math/matrix/log\_softmax.hpp>

8 #include <stan/math/matrix/softmax.hpp>

9

10 namespace stan {

11  namespace agrad {

12

13  template <typename T>

14  inline

15  Eigen::Matrix<fvar<T>,Eigen::Dynamic,1>

16  log\_softmax(const Eigen::Matrix<fvar<T>,Eigen::Dynamic,1>& alpha) {

17  using stan::math::softmax;

18  using stan::math::log\_softmax;

19  using Eigen::Matrix;

20  using Eigen::Dynamic;

21

22  Matrix<T,Dynamic,1> alpha\_t(alpha.size());

23  for (int k = 0; k < alpha.size(); ++k)

24  alpha\_t(k) = alpha(k).val\_;

25

26  Matrix<T,Dynamic,1> softmax\_alpha\_t = softmax(alpha\_t);

27  Matrix<T,Dynamic,1> log\_softmax\_alpha\_t = log\_softmax(alpha\_t);

28

29  Matrix<fvar<T>,Dynamic,1> log\_softmax\_alpha(alpha.size());

30  for (int k = 0; k < alpha.size(); ++k) {

31  log\_softmax\_alpha(k).val\_ = log\_softmax\_alpha\_t(k);

32  log\_softmax\_alpha(k).d\_ = 0;

33  }

34

35  // for each input position

36  for (int m = 0; m < alpha.size(); ++m) {

37  T negative\_alpha\_m\_d\_times\_softmax\_alpha\_t\_m

38  = - alpha(m).d\_ \* softmax\_alpha\_t(m);

39  // for each output position

40  for (int k = 0; k < alpha.size(); ++k) {

41  // chain from input to output

42  if (m == k)

43  log\_softmax\_alpha(k).d\_

44  += alpha(m).d\_

45  + negative\_alpha\_m\_d\_times\_softmax\_alpha\_t\_m;

46  else

47  log\_softmax\_alpha(k).d\_

48  += negative\_alpha\_m\_d\_times\_softmax\_alpha\_t\_m;

49  }

50  }

51

52  return log\_softmax\_alpha;

53  }

54

55

56  }

57 }

58

59 #endif

stan::math::softmax

Eigen::Matrix< T, Eigen::Dynamic, 1 > softmax(const Eigen::Matrix< T, Eigen::Dynamic, 1 > &v)

Return the softmax of the specified vector.

**Definition:** softmax.hpp:46

stan::agrad::fvar

**Definition:** fvar.hpp:13

stan::agrad::log\_softmax

Eigen::Matrix< fvar< T >, Eigen::Dynamic, 1 > log\_softmax(const Eigen::Matrix< fvar< T >, Eigen::Dynamic, 1 > &alpha)

**Definition:** log\_softmax.hpp:16

fvar.hpp

stan::agrad::softmax

Eigen::Matrix< fvar< T >, Eigen::Dynamic, 1 > softmax(const Eigen::Matrix< fvar< T >, Eigen::Dynamic, 1 > &alpha)

**Definition:** softmax.hpp:14

softmax.hpp

Eigen.hpp

log\_softmax.hpp

stan::math::log\_softmax

Eigen::Matrix< T, Eigen::Dynamic, 1 > log\_softmax(const Eigen::Matrix< T, Eigen::Dynamic, 1 > &v)

Return the natural logarithm of the softmax of the specified vector.

**Definition:** log\_softmax.hpp:44

softmax.hpp


---

[ Stan Home Page ]

*© 2011–2014,
Stan Development Team.*
